# Biological Functions of Let-7e-5p in Promoting the Differentiation of MC3T3-E1 Cells

**DOI:** 10.3389/fcell.2021.671170

**Published:** 2021-09-08

**Authors:** Chunli Wang, Songcai Liu, Jiaxin Li, Yunyun Cheng, Zhaoguo Wang, Tianqi Feng, Guanhong Lu, Siyao Wang, Jie Song, Peijun Xia, Linlin Hao

**Affiliations:** ^1^College of Animal Science, Jilin University, Changchun, China; ^2^College of Public Health, Jilin University, Changchun, China

**Keywords:** bone defects, let-7e-5p, osteoblast differentiation, IGF-1, JAK2/STAT5

## Abstract

MicroRNAs let-7c and let-7f, two members of the let-7 family, were involved in regulating osteoblast differentiation and have an important role in bone formation. Let-7e-5p, which also belonged to the let-7 family, presented in the differentiation of adipose-derived stem cells and mouse embryonic stem cells. However, the role of let-7e-5p in osteoblast differentiation was unclear. Thus, this study aimed to elucidate the function of let-7e-5p in osteoblast differentiation and its mechanism. Firstly, we found that the let-7e-5p mimic promoted osteoblast differentiation but not the proliferation of MC3T3-E1 cells by positively regulating the expression levels of osteogenic-associated genes (*RUNX2*, *OCN*, *OPN*, and *OSX*), the activity of ALP, and formation of mineralized nodules. Moreover, we ascertained that the let-7e-5p mimic downregulated the post-transcriptional expression of SOCS1 by specifically binding to the 3′ untranslated region of SOCS1 mRNA. Also, let-7e-5p-induced SOCS1 downregulation increased the protein levels of p-STAT5 and IGF-1, which were both modulated by SOCS1 molecules. Furthermore, let-7e-5p abrogated the inhibition of osteogenic differentiation mediated by SOCS1 overexpression. Therefore, these results suggested that let-7e-5p regulated the differentiation of MC3T3-E1 cells through the JAK2/STAT5 pathway to upregulate IGF-1 gene expression by inhibiting SOCS1. These findings may provide a new insight into the regulatory role of let-7e-5p in osteogenic differentiation and imply the existence of a novel mechanism underlying let-7e-5p-mediated osteogenic differentiation.

## Introduction

Bone repair in trauma, congenital malformations, infections, surgery, and radiotherapy largely depends on the capability of bone formation (Majidinia et al., 2018; Wang T. et al., 2020). Bone formation and resorption are affected by the balance between osteoblast and osteoclast activities, and osteoblast differentiation is a key stage in bone formation that is closely regulated by factors, such as microRNAs (miRNAs) (Dong et al., 2012; Chen et al., 2015; Castano et al., 2020). MiRNAs, which are small non-coding RNA molecules that participate in gene regulation (Ambros, 2004; Bartel, 2004), represent a potential class of bone repair therapeutics because they can enhance the capacity of osteoblast differentiation (Behera and Tyagi, 2018; Chen et al., 2019; Yang et al., 2019; Castano et al., 2020; Won Lee et al., 2020). Numerous miRNAs, including miR-196a, miR-125b, miR-141, miR-29b, miR-335-5p, and let-7, have an essential role in osteoblast differentiation (Mizuno et al., 2008; Itoh et al., 2009; Kim et al., 2009; Li et al., 2009; Zhang et al., 2011). Osteoblasts are the main cells responsible for bone formation through proliferation and differentiation (Salhotra et al., 2020), during which process, the MAPK/ERK (Sun et al., 2015; Lavoie et al., 2020) and PI3K/AKT (Liu et al., 2020; Meng et al., 2021) pathways participate in the regulation of cell proliferation. Type I collagen, osteopontin (*OPN*), osteocalcin (*OCN*), and alkaline phosphatase (ALP) are secreted by cells and have important roles in osteoblast differentiation (Long, 2011). In addition, several signaling pathways, including Wnt/β-catenin (Oh et al., 2020; Park et al., 2020), MAPK (Ge et al., 2007), and JAK/STAT (Levy et al., 1996; Joung et al., 2012, 2013; Darvin et al., 2013; Yu et al., 2018), promote osteogenesis *in vivo* and *in vitro*. Furthermore, the persistent activation of the JAK2/STAT5 signaling pathway increases osteoblastic differentiation (Joung et al., 2012, 2013; Darvin et al., 2013; Yu et al., 2018). Meanwhile, insulin-like growth factor 1 (IGF-1) is regulated by JAK2/STAT5, the downstream signaling pathway of GHR (Darvin et al., 2013). In this process, SOCS1 proteins bind to phosphoserine residues on JAK2 or GHR and then suppress GH signaling by inhibiting JAK2 activity or inducing GHR complex degradation (Pass et al., 2009). IGF-1 promotes bone repair by enhancing bone formation (Blumenfeld et al., 2002; Srouji et al., 2004; He et al., 2006). *In vitro*, IGF-1 is essential for the regulation of osteoblast differentiation (Darvin et al., 2013).

The let-7 family has 10 mature members that are produced from 13 precursor sequences, which are highly conserved across species in terms of sequence and function (Roush and Slack, 2008). An initial small-scale screening attempt using miRNA mimics showed that all let-7 family members can enhance the osteogenic differentiation in human adipose-derived mesenchymal stem cells, suggesting that these miRNAs have similar functions (Wei et al., 2014). Let-7c and let-7f all promote osteogenic differentiation and bone formation (Wei et al., 2014; Shen et al., 2019). However, the function of the other let-7 family members in bone formation has yet to be clarified. Let-7e-5p, which also belonged to the let-7 family, participated in the differentiation of adipose-derived stem cells (Ventayol et al., 2014) and mouse embryonic stem cells (Vinas et al., 2013). Thus, the potential functions of let-7e-5p in bone formation should be elucidated.

Therefore, this study aimed to explore the effect of let-7e-5p on the differentiation of osteoblasts and further clarify the molecular mechanism of this effect by detecting the signaling pathways that involve let-7e-5p. This work is expected to provide a new insight into the regulatory role of let-7e-5p in osteogenic differentiation.

## Materials and Methods

### Cell Culture

The mouse progenitor osteoblast cell line MC3T3-E1 was provided by the Hospital of Stomatology, Jilin University. The MC3T3-E1 cells were cultured in Dulbecco’s minimal essential medium (DMEM, Sigma, United States) supplemented with 10% fetal bovine serum (FBS, Hyclone, United States) and 1% streptomycin/penicillin (Gibco, United States) at 37°C in a humidified 5% CO_2_ incubator. The differentiation medium contained 10 mmol/L β-glycerophosphate (Sigma, United States), 50 mg/mL ascorbic acid, and 10 nM dexamethasone (Sigma, United States) (Wang C. et al., 2020; Wei et al., 2020). HEK293T cells were cultured in DMEM (Hyclone, China) supplemented with 10% FBS and 1% penicillin/streptomycin.

### miRNA and siRNA Transfection

The let-7e-5p mimic; let-7e-5p inhibitor; mimic NC; inhibitor NC; and si-mus-SOCS1, the small interfering RNA used for the knockdown of SOCS1, were synthesized by Genepharma Co., Ltd (Suzhou, China). The sequences of these constructs are shown in Table 1. The nucleotides were transfected with LipoPlus^TM^ Reagent (Sage creation, China) on the basis of the protocol of the manufacturer. MC3T3-E1 cells were seeded on a 6-cm-dish for 24 h before transfection and then transfected with the let-7e-5p mimic, let-7e-5p inhibitor, mimic NC, and inhibitor NC. The cells were then incubated in a complete culture medium or differentiation medium 6 h after transfection.

**TABLE 1:** Sequences of the miRNA oligonucleotides.

Gene	Sequences (5′–3′)
let-7e mimic	Sense: UGAGGUAGGAGGUUGUAUAGUU
	Antisense: CUAUACAACCUCCUACCUCAUU
NC mimic	Sense: UUCUCCGAACGUGUCACGUTT
	Antisense: ACGUGACACGUUCGGAGAATT
let-7e inhibitor	AACUAUACAACCUCCUACCUCA
NC inhibitor	CAGUACUUUUGUGUAGUACAA
si-mus-SOCS1	Sense: ACACUCACUUCCGCACCUUTT
	Antisense: AAGGUGCGGAAGUGAGUGUTT

### Cell Proliferation Assay

Cell Counting Kit-8 (Apexbio, United States) was used for the cell proliferation assay. Briefly, MC3T3-E1 cells were seeded at a density of 5.0 × 10^3^ cells/well into 96-well plates containing 100 μL of culture medium. Then, the cells were cultured for 50 min in a complete culture medium with 10 μL of CCK-8 reagent at 37°C after transfection with the let-7e-5p mimic, let-7e-5p inhibitor, mimic NC, and inhibitor NC at 0, 24, 48, 72, and 96 h. Absorbance at a wavelength of 450 nm was measured by using a microplate reader (TECAN, Switzerland).

### RNA Extraction and Quantitative Real-Time PCR

Total RNA was isolated from the cells by using the RNAiso Plus reagent (Takara, United States) in accordance with the instructions of the manufacturer. Then, cDNA was generated with a reverse transcription kit. An ABI PRISM 7900HT thermocycler (Applied Biosystems, United States) was used for quantitative real-time PCR (qRT-PCR) with SYBR Select Master Mix (Roche). The primer sequences are displayed in Table 2. The relative expression levels of let-7e-5p and other genes were normalized against those of small nuclear RNA U6 and β-actin, respectively, by using the 2^–ΔΔCt^ method.

**TABLE 2:** Primer information for qRT-PCR analysis of expression of target genes.

Genes	Sequences (5′–3′)	Product lengths (bp)	Tm (°C)
*COL1a1*	F: CCAGCCGCAAAGAGTCTACA R: TTCCACGTCTCACCATTGGG	170	58.5
*OPN*	F: ACACTTTCACTCCAATCGTCC R: TGCCCTTTCCGTTGTTGTCC	240	58.5
*RUNX2*	F: GAGGGACTATGGCGTCAAACA R: GGATCCCAAAAGAAGCTTTGC	70	58.5
*OSX*	F: TCAGCCGCCCCGATCTTCCA R: CAATGGGTCCACCGCGCCAAG	157	58.5
*OCN*	F: AGACTCCGGCGCTACCTT R: CTCGTCACAAGCAGGGTTAG	93	58.5
*IGF-1*	F: TGGTGGATGCTCTTCAGTTCGT R: TGCTTTTGTAGGCTTCAGTGGG	179	58.5
β-*actin*	F: GGCTGTATTCCCCTCCATCG R: CCAGTTGGTAACAATGCCATGT	154	58.5
let-7e-5p	F: GGCCTGAGGTAGGAGGTTGT R: CAGTGCGTGTCGTGGAGT	66	58.5
*U6*	F: CTCGCTTCGGCAGCACA R: AACGCTTCACGAATTTGCG	71	58.5

### Western Blot Analysis

A BCA protein assay kit (KeyGEN BioTECH, China) was applied to detect protein concentrations. Western blot assays were performed as previously reported (Wang C. et al., 2020). In this study, antibodies against AKT (1:2000, CST, United States), phosphor-AKT (1:2000, CST, United States), ERK1/2 (1:2000, CST, United States), and phosphor-ERK1/2 (1:2000, CST, United States) were used after 48 h of transfection to investigate the effects of let-7e-5p on MC3T3-E1 cell proliferation. For the evaluation of the effect of let-7e-5p on target gene expression, the antibody against SOCS1 (1:1000, Abcam, England) was used after 48 h of transfection. Antibodies against phosphor-STAT5 (1:1,000, Abcam, England), STAT5 (1:2,000, Abcam, England), OPN (1:500, WanLeiBio, China), collagen I (1:500, WanLeiBio, China), phosphor-JAK2 (1:500, WanLeiBio, China), and JAK2 (1:500, WanLeiBio, China) were used after 72 h of differentiation culture to evaluate the function of let-7e-5p in the osteogenic differentiation of MC3T3-E1 cells. The protein quantities detected by each antibody were analyzed by using the GenoSens gel analysis software and normalized on the basis of β-actin (1:2,000, BBI, China).

### Measurement of ALP Activity

Confluent MC3T3-E1 cells were transfected with the let-7e-5p mimic, let-7e-5p inhibitor, and NC in 24-well plates. Six hours after transfection, the cells were incubated in differentiation media and finally collected on 3, 5, 7, 14, and 21 days post-transfection. An ALP kit (Nanjing Jiancheng, China) was utilized to measure ALP activities in accordance with the instructions of the manufacturer. A BCA protein assay kit (Beyotime, China) was used to detect the protein content on the basis of the protocols of the manufacturer. A microplate reader (TECAN, China) was applied to detect absorbance at 520 nm for the determination of enzyme activity. ALP activity was normalized by using the total protein content.

### Alizarin Red Staining

Mineralization is the last stage of osteogenic differentiation, and the formation of mineralized nodules is a crucial marker of osteoblast mineralization (Wang C. et al., 2020). The chelation of Alizarin Red (AR) with the calcium ions that form mineralized nodules is used to determine the osteogenic mineralization capability in tissues or cells (Puchtler et al., 1969; Xi et al., 2016; Zheng et al., 2018). Confluent MC3T3-E1 cells were transfected with the let-7e-5p mimic, let-7e-5p inhibitor, and negative controls in a six-well plate. Six hours after transfection, the cells were cultured in differentiation media for 21 days. Then, AR staining (ARS) was performed as previously reported (Wang C. et al., 2020). The results were replicated in at least three independent experiments.

### Bioinformatics Analysis

TargetScan^1^ and miRanda^2^ were applied to predict the target of let-7e-5p. In this research, *SOCS1*, one of the targets, was selected as the candidate. The secondary structure formed by the binding of let-7e-5p to the 3′ untranslated region (UTR) of the *SOCS1* gene, and the minimum free energy (MFE) value were predicted by using mfold.^3^

### Plasmid Construction and Transfection

In accordance with the seed sequence of let-7e-5p, the complementary oligonucleotides of the wild-type (WT) or seed-mutated (MUT) 3′UTR of the *SOCS1* gene were synthesized by Genewiz (Tianjin, China) and inserted into a psiCHECK^TM^2 vector (Promega, United States) to construct the recombinant vectors. The constructs were named *SOCS1*-WT and *SOCS1*-MUT.

The full coding sequence of SOCS1 was amplified via PCR by using primers (forward 5′CGCGGATCCCTC GAGTAGGATGGTAGCACG3′; reverse 5′ CCGGATATCTCAA ATGAAGCCAGAGACCCTCC3′) and cloned into the pcDNA3.1 expression vector with *BamH*I and *EcoR*V. The SOCS1 overexpression vector was named pcDNA3.1-SOCS1. MC3T3-E1 cells were seeded onto a 6-cm dish or 24-well plates for 24 h before transfection to study the effect of SOCS1 in osteoblast differentiation. Then, pcDNA3.1, pcDNA3.1-SOCS1, the mixtures of pcDNA3.1 and mimic NC, let-7e-5p mimic and pcDNA3.1, or let-7e-5p mimic and pcDNA3.1-SOCS1 were transfected into MC3T3-E1 cells with the LipoPlus^TM^ Reagent (Sage Creation, China). Six hours after transfection, the cells were incubated in a differentiation medium.

### Dual-Luciferase Reporter Assay

HEK-293T cells were plated on 96-well culture plates at a density of 1 × 10^4^ cells/well and cotransfected with *SOCS1*-WT and *SOCS1*-MUT and miRNA NC or mimic duplexes (10 pM) for 48 h with the LipoPlus^TM^ Reagent (Sage creation, China). The cells were harvested, and luciferase activities were detected by the means of a Dual-Luciferase Reporter Assay system (TECAN, Switzerland) in accordance with the instructions of the manufacturer.

### Statistics Analysis

All experimental results were shown as mean ± S.E.M with at least three independent replications. One-way and two-way ANOVA were applied to test the statistical differences among different groups. All statistical analyses were performed by using the GraphPad Prism 6.0. Here, *p* < 0.05 was regarded as statistically significant (^∗^*p* < 0.05, ^∗∗^*p* < 0.01).

## Results

### Let-7e-5p Did Not Affect the Proliferation of MC3T3-E1 Cells

**FIGURE 1:**
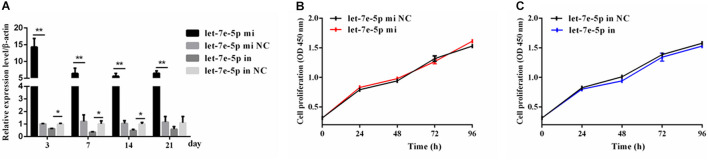
Let-7e-5p had no significant effect on MC3T3-E1 cell proliferation. **(A)** qRT-PCR detection of the transfection efficiency of the let-7e-5p mimic or inhibitor in MC3T3-E1 cells on days 3, 7, 14, and 21. **(B,C)** Proliferation of MC3T3-E1 cells transfected with the let-7e-5p mimic **(B)** or inhibitors **(C)** (*p < 0.05; **p < 0.01).

**FIGURE 2:**
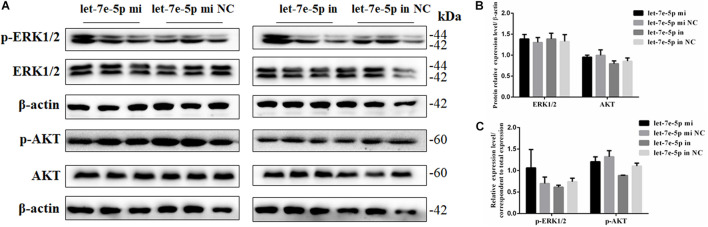
Let-7e-5p had no significant effect on proliferation-related signal pathways. **(A)** Western blot analysis results for p-ERK1/2, ERK1/2, p-AKT, and AKT protein expression following transfection with the let-7e-5p mimic or inhibitor. **(B,C)** Quantification of Western blot analysis results. Data are presented as the mean ± S.E.M of three lanes of the panel (^∗^*p* < 0.05).

Given that previous studies have shown that let-7e-5p has an important effect on the proliferation of many types of cancer cells (Lin et al., 2017; Li et al., 2018; Wang et al., 2019), the let-7e-5p mimic and inhibitor were first transfected into MC3T3-E1 cells, and cell proliferation was then detected. As expected, in the transfected MC3T3-E1 cells, the let-7e-5p mimic markedly increased the let-7e-5p levels compared with the mimic NC (*p* < 0.01), whereas the let-7e-5p inhibitor reduced the let-7e-5p levels compared with the inhibitor NC (*p* < 0.05) (Figure 1A). Furthermore, the increase and decrease in the let-7e-5p expression continued for 21 days (Figure 1A). Cell proliferation did not significantly differ after let-7e-5p mimic transfection (Figures 1B,C) (*p* > 0.05). Possible signaling pathways (MAPK/ERK and PI3K/AKT signaling pathways) were detected via Western blot analysis to further explore the effect of let-7e-5p on cell proliferation. The results demonstrated that the phosphorylation of AKT and ERK1/2 in the let-7e-5p mimic group did not significantly differ from that in the mimic NC group (Figure 2) (*p* > 0.05), suggesting that let-7e-5p did not enhance the growth potential of MC3T3-E1 cells.

### Let-7e-5p Promoted the Osteogenic Differentiation of MC3T3-E1 Cells

**FIGURE 3:**
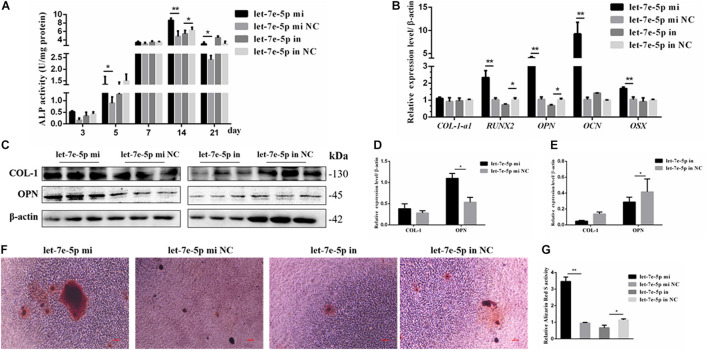
Let-7e-5p promoted the differentiation of MC3T3-E1 cells. **(A)** Effect of let-7e-5p on ALP activity in MC3T3-E1 cells on days 3, 5, 7, 14, and 21. **(B)** Effect of let-7e-5p on the mRNA levels of osteogenic-differentiation-related genes in MC3T3T-E1 cells. **(C)** Western blot analysis of COL-1 and OPN protein levels in MC3T3-E1 cells. **(D,E)** Columnar charts of **(C)** after grayscale analyses. Data are presented as the mean ± S.E.M of the quantified values of three lanes (*p < 0.05). **(F)** Effect of let-7e-5p on the mineralization of MC3T3-E1 cells on day 21. Images were captured with an inverted phase microscope (scale bar, 100 μm). **(G)** Mineralization results quantified with 10% cetylpyridinium chloride (**p < 0.01).

The effect of let-7e-5p on osteoblast differentiation was further detected. ALP activity was used to assess the effect of let-7e-5p on cell differentiation because it is regarded as a marker of early osteogenic differentiation (Bancroft et al., 2002; Hosseini et al., 2019). In brief, the let-7e-5p mimic and inhibitor were first transfected into the MC3T3-E1 cells. The cells were then cultured in a differentiation medium, and ALP activities were separately measured on days 3, 5, 7, 14, and 21. The results showed that let-7e-5p significantly enhanced ALP activities compared with the mimic NC on day 5 (*p* < 0.05), day 14 (*p* < 0.01), and day 21 (*p* < 0.05); this effect was counteracted on day 14 by the let-7e-5p inhibitor (Figure 3A) (*p* < 0.05). The expression of osteogenic-differentiation-related genes in each group on day 3 after differentiation culture was detected. Comparison with the let-7e-5p mimic NC showed that the let-7e-5p mimic did not significantly influence the mRNA expression of collagen type I, alpha 1 (*COL-1-α1*) (Figure 3B) (*p* > 0.05), and instead significantly increased the mRNA expression of runt-related transcription factor 2 (*RUNX2*), *OPN*, *OCN*, and osterix (*OSX*) (Figure 3B) (*p* < 0.01). By contrast, the knockdown of let-7e-5p reduced *OPN* and *RUNX2* expression (Figure 3B) (*p* < 0.05). The protein levels of COL-1 and OPN were further detected via Western blot analysis considering their vital roles in osteoblast differentiation (Chai et al., 2019; Hosseini et al., 2019). The results revealed that *COL-1* was expressed at slightly higher levels in the let-7 mimic group than in the let-7e-5p mimic NC group (*p* > 0.05) (Figures 3C,D) and that the expression levels of *OPN* were significantly higher in the let-7 mimic group than in the let-7e-5p mimic NC group (Figures 3C,D) (*p* < 0.05). However, the knockdown of let-7e-5p reduced *OPN* expression (Figures 3C,E) (*p* < 0.05). In accordance with these results, ARS demonstrated that the staining intensity of mineralization nodes was markedly increased in the let-7e-5p mimic group (*p* < 0.01) and reduced in the let-7e-5p inhibitor group (Figures 3F,G) (*p* < 0.05). The consistency of these results with the results presented in the previous section indicated that let-7e-5p contributed a positive regulation potential to osteoblast differentiation.

### Let-7e-5p Directly Targeted the *SOCS1* 3′UTR and Inhibited *SOCS1* Expression

**FIGURE 4:**
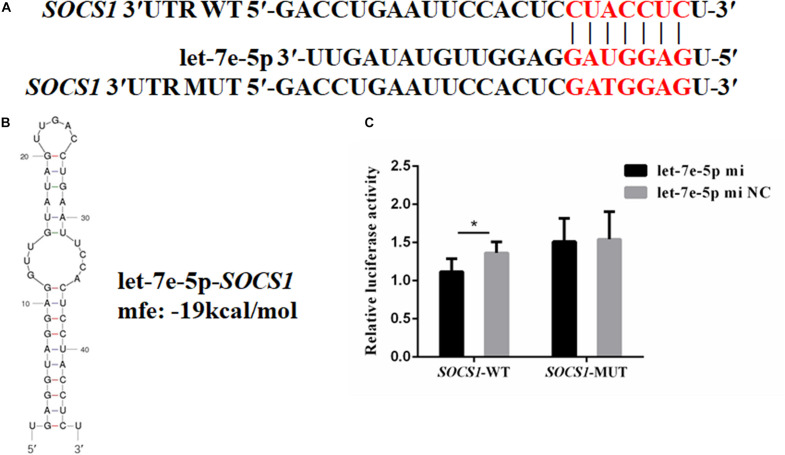
*SOCS1* was the direct target gene of let-7e-5p. **(A)** Seed regions of let-7e-5p and the seed-recognizing sites in the 3′UTR (WT and MUT) of the *SOCS1* gene. The seed-recognizing sites were marked in red font. **(B)** RNA secondary structure and MFE formed by the binding of let-7e-5p to the 3′UTR of the *SOCS1* gene predicted by mfold and RNAhybrid. **(C)** Relative luciferase activities in HEK-293T cells cotransfected with the constructed *SOCS1*-WT and *SOCS1*-MUT vectors and the let-7e-5p mimic or let-7e-5p mimic NC. (*p < 0.05).

**FIGURE 5:**
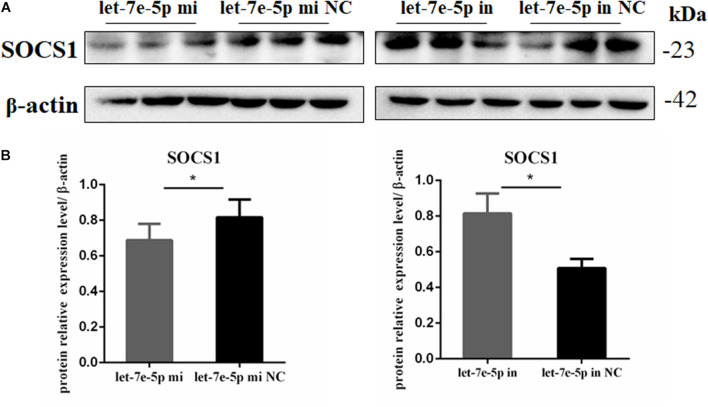
Let-7e-5p reduced the expression of *SOCS1*. **(A)** Western blot analysis results for SOCS1 expression in MC3T3-E1 cells transfected with the let-7e-5p mimic or inhibitor (*p < 0.05). **(B)** Quantification of Western blot analysis results. Data are presented as the mean ± S.E.M of the quantified values of three lanes (*p < 0.05).

TargetScan and RNAhybrid were used to predict and screen the let-7e-5p target genes to determine the potential target through which let-7e-5p exerts its effect on MC3T3-E1 cells. *SOCS1*, which is involved in the JAK2/STAT5 pathway, was selected as the candidate target in this research. The predictive software showed that the *SOCS1* 3′UTR contained putative binding sites for the let-7e-5p seed sequence (Figure 4A). The MFE formed by let-7e-5p and the *SOCS1* gene was 19.0 kcal/mol. The secondary structure formed by the binding of let-7e-5p to the 3′UTR of the *SOCS1* gene is shown in Figure 4B. The *SOCS1*-WT or *SOCS1*-MUT vectors and the let-7e-5p mimic or let-7e-5p mimic NC were cotransfected into HEK293T cells. The results showed that the luciferase activity of the cells transfected with the let-7e-5p mimic and *SOCS1* 3′UTR was markedly reduced compared with that of the cells cotransfected with the let-7e-5p mimic NC and *SOCS1* 3′UTR (*p* < 0.05) (Figure 4C). These results were consistent with previous findings showing that *SOCS1* is a direct target gene of let-7e-5p in RAW264.7 cells (Li et al., 2021). Then, we explored the effect of the let-7e-5p mimic on the expression of the SOCS1 protein. Western blot analysis revealed that in contrast to the let-7e-5p mimic NC, the let-7e-5p mimic reduced SOCS1 protein levels in MC3T3-E1 cells; this effect was counteracted by the inhibitor (Figures 5A,B) (*p* < 0.05). This result was consistent with the relative luciferase activity measurements obtained in this work and in a previous study (Li et al., 2021).

### SOCS1 Inhibited the Osteogenic Differentiation of MC3T3-E1 Cells

**FIGURE 6:**
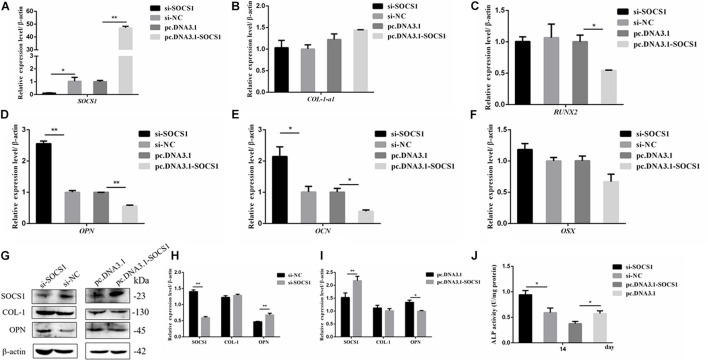
SOCS1 suppressed the differentiation of MC3T3-E1 cells. **(A)** SOCS1 mRNA level after SOCS1 overexpression and knockdown. **(B–F)** Effect of SOCS1 on ALP activity and mRNA amounts of differentiation-related genes. **(G)** Western blot analysis of COL-1 and OPN protein levels in MC3T3-E1 cells. **(H,I)** Columnar charts of **(G)** after grayscale analyses. Data are presented as the mean ± S.E.M of the values of three lanes (*p < 0.05). **(J)** Effect of SOCS1 on ALP activity in MC3T3-E1 cells on day 14. (*p < 0.05; **p < 0.01).

The efficiencies of the overexpression plasmid (pcDNA3.1-SOSC1) and si-SOCS1 were detected via qRT-PCR and Western blot analyses. The results confirmed that SOCS1 was successfully overexpressed and knocked-down in MC3T3-E1 cells (Figures 6A,G). The expression of osteogenic-differentiation-related genes in each group on day 3 after differentiation culture was detected. The results showed that compared with the si-NC, si-SOCS1 did not significantly influence the mRNA expression levels of *RUNX2*, *OSX*, and *COL-1-a1* (Figures 6B,C,F) (*p* > 0.05) but significantly increased the mRNA expression levels of *OCN* and *OPN* (Figures 6D,E) (*p* < 0.01). Furthermore, compared with pc.DNA3.1, pc.DNA3.1-SOCS1 significantly reduced the mRNA expression levels of *RUNX2*, *OCN*, and *OPN*. The protein levels of COL-1 and OPN were further detected through Western blot analysis due to their vital roles in osteoblast differentiation (Chai et al., 2019; Hosseini et al., 2019). The results revealed that *COL-1* expression in the si-SOCS1 and pc.DNA3.1-SOCS1 groups had slightly changed compared with that in the si-NC and pc.DNA3.1 groups (*p* > 0.05). OPN protein expression was inhibited by SOCS1 overexpression and upregulated after SOCS1 knockdown (Figures 6G–I) (*p* < 0.05). Also, the activity of ALP in MC3T3-E1 cells on day 14 was inhibited by SOCS1 overexpression and promoted after SOCS1 knockdown (Figure 6J) (*p* < 0.05). These results suggested that SOCS1 could serve as an inhibitor of osteoblast differentiation.

### Let-7e-5p Regulated the JAK2/STAT5 Signal Pathway and IGF-1 Expression

**FIGURE 7:**
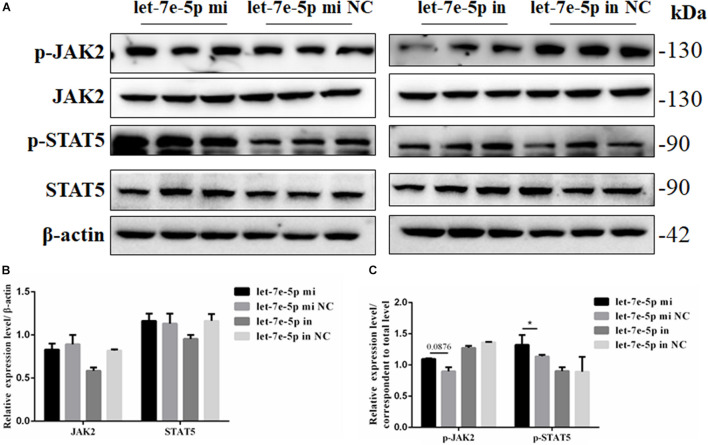
Let-7e-5p activated the differentiation-related signal pathway. **(A)** Western blot analysis results for p-JAK2, JAK2, p-STAT5, and STAT5 protein expression following transfection with the let-7e-5p mimic or inhibitor. **(B,C)** Quantification of the Western blot analysis results (*p < 0.05).

Considering the effect of let-7e-5p on osteogenic differentiation, the possible signaling pathways (JAK2/STAT5 signaling pathways) involved in this effect were examined to investigate how let-7e-5p regulated the differentiation of MC3T3-E1 cells. The results demonstrated that compared with the let-7e-5p mimic NC, the let-7e-5p mimic significantly increased the phosphorylation of STAT5 (*p* < 0.05) but did not obviously increase the phosphorylation of JAK2 (Figure 7) (*p* > 0.05). These results suggested that let-7e-5p regulated osteogenic differentiation, at least partially, through the JAK2/STAT5 signaling pathway.

**FIGURE 8:**
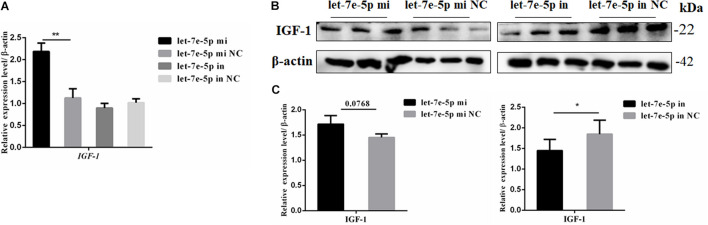
Let-7e-5p decreased the expression of *IGF-1*. **(A)** mRNA level of *IGF-1* after transfection with the let-7e-5p mimic or inhibitor. **(B)** *IGF-1* expression in MC3T3-E1 cells was determined via Western blot analysis after transfection with the let-7e-5p mimic and inhibitor. **(C)** Quantification of the Western blot analysis results (*p < 0.05; **p < 0.01).

IGF-1 is regulated as a typical downstream target of STAT5 (Darvin et al., 2013). The mRNA and protein expression levels of *IGF-1* were further detected through the transfection of the let-7e-5p mimic and inhibitor into MC3T3-E1 cells. The results showed that compared with the let-7e-5p mimic NC, the let-7e-5p mimic significantly increased the expression of *IGF-1* mRNA (Figure 8A) (*p* < 0.05). Although the Western blot analysis revealed that *IGF-1* protein expression was not obvious, the same trend was identified (Figures 8B,C) (*p* = 0.0768). These effects were counteracted by the inhibitors (Figures 8B,C) (*p* < 0.05).

### SOCS1 Inhibited the Let-7e-5p-Induced Osteogenic Differentiation of MC3T3-E1 Cells

**FIGURE 9:**
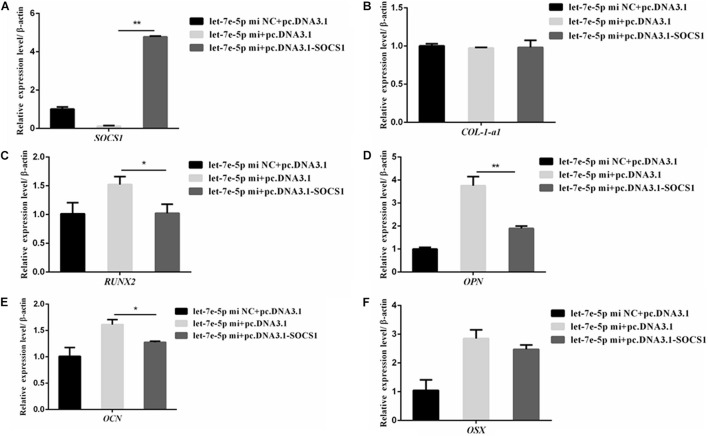
SOCS1 inhibited let-7e-5p-induced osteogenic differentiation in MC3T3-E1 cells. **(A)** mRNA level of *SOCS1* after transfection with the let-7e-5p mimic NC and pc.DNA3.1, let-7e-5p mimic and pc.DNA3.1, or let-7e-5p mimic and pc.DNA3.1-SOCS1. **(B–F)** mRNA levels of osteogenic-differentiation-related genes after transfection with the let-7e-5p mimic NC and pc.DNA3.1, let-7e-5p mimic and pc.DNA3.1, or let-7e-5p mimic and pc.DNA3.1-SOCS1 (*p < 0.05; **p < 0.01).

SOCS1 overexpression by using an expression vector was confirmed via qRT-PCR (Figure 9A) to further confirm that SOCS1 was involved in a let-7e-5p-induced osteoblast differentiation. Subsequently, MC3T3-E1 cells were cotransfected with the let-7e-5p mimic-NC and pc.DNA.3.1, let-7e-5p mimic and pc.DNA.3.1, or let-7e-5p mimic and pc.DNA.3.1-SOCS1 expression vector. The results showed that compared with the let-7e-5p mimic and pc.DNA.3.1, the let-7e-5p mimic and pc.DNA.3.1-SOCS1 did not significantly influence the expression of *OSX* and *COL-1-a1* mRNA (Figures 9B,F) (*p* > 0.05) but instead significantly decreased the expression of *RUNX2*, *OCN*, and *OPN* mRNA and *OPN* protein (Figures 9C–E) (*p* < 0.01). As a result, the overexpression of SOCS1 inhibited the let-7e-5p-induced osteogenic differentiation of MC3T3-E1 cells. All these results further indicated that let-7e-5p mediated the induction of osteogenic differentiation by targeting SOCS1.

## Discussion

In recent years, miRNAs have been regarded as the pivotal regulators that participate in bone repair and regeneration caused by trauma, congenital malformations, infections, surgery, and radiotherapy (Zhang et al., 2017; Chen et al., 2019; Hosseinpour et al., 2019; Zhai et al., 2020). During these processes, many miRNAs exert a considerable effect on osteoblast differentiation and bone formation by increasing the activity of ALP and regulating the expression of genes related to osteogenic differentiation (*COL-1-α1*, *OPN*, *OCN*, *RUNX2*, and *OSX*) (Itoh et al., 2009; Kim et al., 2009; Li et al., 2009). The let-7 family, which consists of several members, has an important influence on various cellular activities, including the committed differentiation of multiple cell types (Wulczyn et al., 2007; Boyerinas et al., 2010; Eguchi et al., 2013). An initial small-scale screening study involving miRNA mimics indicated that all the members of the let-7 family can enhance the osteogenic differentiation of hADSCs, thus demonstrating that these miRNAs have similar functions; for example, let-7c promotes the osteogenic differentiation of hADSCs by increasing RUNX2, OSX, and ALP, and let-7f can rescue the Dex-inhibited osteogenic differentiation of murine BMSCs (Wei et al., 2014; Shen et al., 2019). Let-7e-5p is involved in the differentiation of adipose-derived stem cells (Ventayol et al., 2014) and mouse embryonic stem cells (Vinas et al., 2013), However, the effect and mechanism of let-7e-5p in osteogenic differentiation have yet to be clarified. We suspected that let-7e-5p likely plays a vital role in regulating the differentiation of osteoblasts and then participates in bone formation. The activity of ALP and the expression levels of genes related to osteogenic differentiation (*RUNX2*, *OPN*, *OCN*, and *OSX*) regulate osteoblast differentiation and bone formation in growing bones; the expression levels of the above genes gradually increase during osteoblast differentiation and are important factors for evaluating cell differentiation (Wang C. et al., 2020; Wei et al., 2020). Similar to the results of previous works, our results for the transfection of the let-7e-5p mimic confirmed that let-7e-5p enhanced the osteoblast differentiation potential by increasing the ALP activity (Xi et al., 2016) and regulating *RUNX2*, *OCN*, *OPN*, and *OSX* expression in MC3T3-E1 cells. The above results further verified that these miRNAs have similar functions (Roush and Slack, 2008). Further studies involving animal models are warranted to prove this conclusion.

The differentiation potential of osteoblasts is subject to various internal and external factors that are regulated by signaling pathways. These signaling pathways include the Wnt/β-catenin (Oh et al., 2020; Park et al., 2020), MAPK (Ge et al., 2007), and JAK/STAT (Levy et al., 1996; Joung et al., 2012, 2013; Darvin et al., 2013; Yu et al., 2018) pathways, which have all been reported to promote osteogenesis *in vivo* and *in vitro*. Furthermore, let-7 participates in the JAK2/STAT signaling pathway in cancer cells by targeting SOCS4 and activates the JAK1/STAT3 signaling pathway (Patel et al., 2014; Chen et al., 2017). Meanwhile, SOCS1 proteins participate in the JAK2/STAT signaling pathway by inhibiting JAK2 activity or inducing GHR complex degradation (Pass et al., 2009). The potential target gene of let-7e-5p was predicted via bioinformatics analysis to further explore the regulatory mechanism of let-7e-5p in osteogenic differentiation. The *SOCS1* gene, which is involved in the JAK2/STAT5 pathway, was predicted to be targeted by let-7e-5p. This result was further confirmed through an analysis with the dual luciferase reporter system and was consistent with the findings of Wu et al., who found that *SOCS-1* is the direct target gene of let-7e-5p (Li et al., 2021). A growing number of studies have revealed that *SOCS1* negatively affects the downstream JAK2/STAT5 signaling pathway (Oh et al., 2009; Zhao et al., 2016; Liau et al., 2018; Zhang X. H. et al., 2018), and the reduction in SOCS1 expression enhances the expression of p-JAK2 and p-STAT5 (Zhao et al., 2016; Zhang X. H. et al., 2018). Furthermore, the JAK2/STAT5B pathway enhances the osteoblast differentiation of osteoblast-like cells and MSCs (Joung et al., 2012, 2013). In this study, we found that SOCS1 could suppress the differentiation of MC3T3-E1 cells by inhibiting *RUNX2*, *OCN*, and *OPN* expression and ALP activity. Meanwhile, transfection with the let-7e-5p mimic activated the JAK2/STAT5 pathway, suggesting that the activation of this pathway was related to let-7e-5p-regulated osteoblast differentiation. This result was consistent with the capability of let-7e-5p to reduce the expression of SOCS1. Moreover, the JAK2/STAT5 signaling pathway has considerable effects on the expression of various differentiation markers (Darvin et al., 2013; Yu et al., 2018), and the activation of the JAK2/STAT5 signaling pathway promotes the expression of IGF-1 (Moon et al., 2015; Zhang M. et al., 2018). The administration of IGF-1, a typical downstream target of STAT5, with nanoparticles to promote osteogenesis and angiogenesis, which play a pivotal role in the regulation of bone formation, has become increasingly practiced (Ishida et al., 2010; Zhang et al., 2020). IGF-1 treatment almost completely rescues all of the effects of GHR^(–/–)^ on bone growth and remodeling, suggesting that IGF-1 directly affects the osteoblasts (Sims et al., 2000). As expected, our present findings demonstrated that let-7e-5p regulated osteogenic differentiation by inhibiting SOCS1. This effect resulted in the activation of the JAK2/STAT5 signaling pathway and then in the upregulation of IGF-1 expression.

In conclusion, let-7e-5p participated in osteogenic differentiation by mediating SOCS1 expression likely by promoting the JAK2/STAT5 signaling pathway activation and IGF-1 expression. Further studies involving animal models are warranted to prove this conclusion. The results of the present study may provide a new insight into the regulatory role of let-7e-5p in osteogenic differentiation.

## Data Availability Statement

The original contributions presented in the study are included in the article/supplementary material, further inquiries can be directed to the corresponding authors.

## Author Contributions

LH, CW, and SL conceived and designed the experiments. CW performed the experiments. CW and SL wrote the manuscript. All authors assessed the experiments, provided the data analysis, contributed to the manuscript, and approved the submitted version.

## Conflict of Interest

The authors declare that the research was conducted in the absence of any commercial or financial relationships that could be construed as a potential conflict of interest.

## Publisher’s Note

All claims expressed in this article are solely those of the authors and do not necessarily represent those of their affiliated organizations, or those of the publisher, the editors and the reviewers. Any product that may be evaluated in this article, or claim that may be made by its manufacturer, is not guaranteed or endorsed by the publisher.

## Footnotes

1
http://www.targetscan.org

2
http://www.microrna.org

3
http://unafold.rna.albany.edu
